# Modulation of mammalian translation by a ribosome-associated tRNA half

**DOI:** 10.1080/15476286.2020.1744296

**Published:** 2020-03-30

**Authors:** Yulia Gonskikh, Matthias Gerstl, Martin Kos, Nicole Borth, Markus Schosserer, Johannes Grillari, Norbert Polacek

**Affiliations:** a Department of Chemistry and Biochemistry, University of Bern, Bern, Switzerland; b Graduate School for Cellular and Biomedical Sciences, University of Bern, Bern, Switzerland; c Department of Biotechnology, BOKU - University of Natural Resources and Life Sciences, Vienna, Austria; d Biochemistry Center, University of Heidelberg, Heidelberg, Germany; e Christian Doppler Laboratory on Biotechnology of Skin Aging, Vienna, Austria; f Ludwig Boltzmann Institute for Experimental and Clinical Traumatology, Vienna, Austria

**Keywords:** tRNA-derived fragments, tRNA halves, ncRNA, rancRNA, translation regulation, ribosome

## Abstract

Originally considered futile degradation products, tRNA-derived RNA fragments (tdRs) have been shown over the recent past to be crucial players in orchestrating various cellular functions. Unlike other small non-coding RNA (ncRNA) classes, tdRs possess a multifaceted functional repertoire ranging from regulating transcription, apoptosis, RNA interference, ribosome biogenesis to controlling translation efficiency. A subset of the latter tdRs has been shown to directly target the ribosome, the central molecular machine of protein biosynthesis. Here we describe the function of the mammalian tRNA^Pro^ 5ʹ half, a 35 residue long ncRNA associated with ribosomes and polysomes in several mammalian cell lines. Addition of tRNA^Pro^ halves to mammalian *in vitro* translation systems results in global translation inhibition and concomitantly causes the upregulation of a specific low molecular weight translational product. This tRNA^Pro^ 5ʹ half-dependent translation product consists of both RNA and amino acids. Transfection of the tRNA^Pro^ half into HeLa cells leads to the formation of the same product *in vivo*. The migration of this product in acidic gels, the insensitivity to copper sulphate treatment, the resistance to 3ʹ polyadenylation, and the association with 80S monosomes indicate that the accumulated product is peptidyl-tRNA. Our data thus suggest that binding of the tRNA^Pro^ 5ʹ half to the ribosome leads to ribosome stalling and to the formation of peptidyl-tRNA. Our findings revealed a so far unknown functional role of a tdR thus further enlarging the functional heterogeneity of this emerging class of ribo-regulators.

## Introduction

In early high-throughput studies, tRNA-derived fragments (tdRs) were identified in a wide variety of model systems yet they were considered as degradation products devoid of physiological relevance [1]. Nowadays, with the improved deep-sequencing protocols, tailor-made bioinformatics tools, and accumulating functional studies it became clear that tdRs represent a so far unknown class of regulatory ncRNAs. It could be demonstrated that many tdRs, which include tRNA half molecules as well as even shorter tRNA fragments (tRFs), are specifically processed by endonucleases to participate in and regulate diverse biological process [2]. tRNA halves and tRNA fragments are involved in controlling cell proliferation, cellular homoeostasis, priming of viral reverse transcriptases, regulating gene expression, trans-generational epigenetic inheritance, RNA processing, modulation of the DNA damage response, tumour suppression, regulation of transposition, neurodegeneration, ribosome biogenesis, and translation regulation [2–5]. Accumulating evidence further link misregulated tRF production to certain human diseases, while other studies highlight tRFs as potential biomarkers for disease states [6–8].

Recently, ribosome-associated non-coding RNAs (rancRNAs) were recognized as a new class of regulatory ncRNAs. It was shown that small rancRNAs influence protein synthesis by directly targeting the ribosome in organisms representing all three domains of life [9]. A major fraction of these rancRNAs is processed from longer functional precursors including mRNAs, snoRNAs, and tRNAs [10,11]. Functional analyses of selected ribosome-bound tRFs revealed an unexpected functional heterogeneity. For example, an alkaline stress-induced archaeal tRNA^Val^ fragment associates with the small ribosomal subunit and globally inhibits translation initiation by competing with mRNA binding [12,13]. Another study suggests that yeast ribosome-bound tRFs have the potential of inhibiting tRNA aminoacylation [14]. On the other hand, in the human parasite *T. brucei* a tRNA^Thr^ 3ʹ half associates with ribosomes under nutrient deprivation conditions and stimulates global translation during stress recovery [15]. Ribosome-targeted tdRs have recently also been linked to cell proliferation in mammalian cells and might promote cancer progression in a mouse model [16]. These findings reveal ribosome-bound tdRs as functionally versatile regulators capable of fine-tuning protein biosynthesis in a stress-dependent manner.

Despite recent studies highlighting regulatory roles during gene expression in different model organisms, rancRNA has not yet been studied in detail in human cells or any other mammalian species so far. In this study, we performed screening for ribosome-bound tRFs in different mammalian cell lines and uncovered the highly conserved tRNA^Pro^ 5ʹ half. We demonstrate that upon ribosome-binding the tRNA^Pro^ 5ʹ half inhibits global protein biosynthesis with the concomitant appearance of a low molecular weight translation product, dubbed ProTiP. Cumulative experimental evidence suggests ProTiP to be peptidyl-tRNA likely accumulating as a by-product of tRNA^Pro^ half-mediated translation inhibition.

## Results

### Expression and ribosome-association of the tRNA^Pro^ half in mammalian cell lines

**Figure 1.**
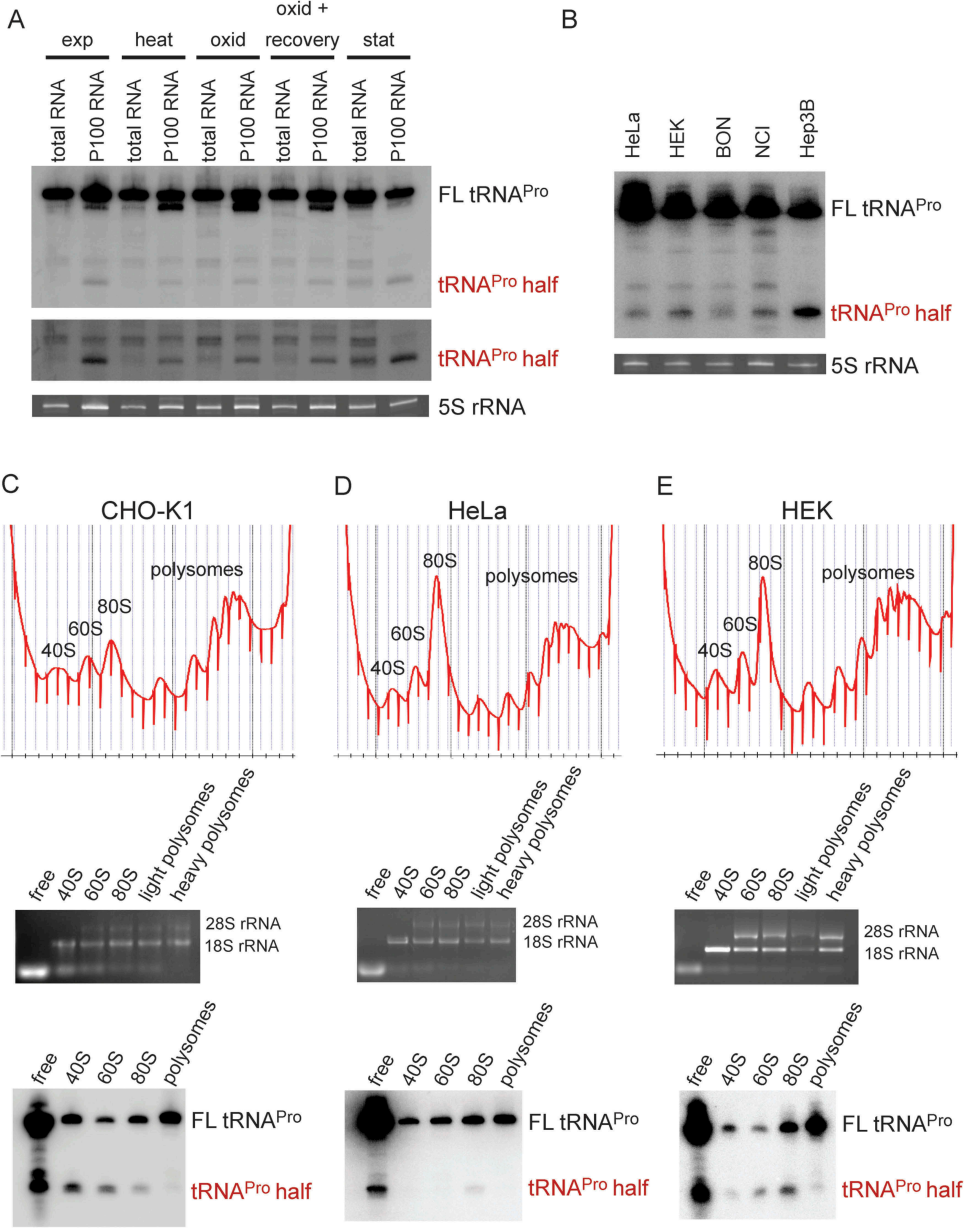
Expression and ribosome association of the tRNA^Pro^ half in different mammalian cell lines. (A) Northern blot analysis of the tRNA^Pro^ 5ʹ half of 20 µg of total RNA or 20 µg of RNA isolated from the crude ribosome pellet (P100) from CHO-K1 at several stress conditions: heat shock (heat), oxidative stress (oxid), oxidative stress with recovery, stationary (stat) growth phase and as a control exponential (exp) growth phase. Lower panel shows the same northern blot in the region of the tRNA half with increased contrast. (B) Northern blot analysis of the tRNA^Pro^ 5ʹ half of 30 µg total RNA isolated from HeLa, HEK, BON, NCI, and Hep3B cells. Ethidium bromide-stained 5 S rRNA serves as a loading control. (C-E) Polysome profiling of exponentially grown CHO-K1, HeLa, and HEK cells, respectively (top panels). RNA was isolated from the indicated fractions (free RNA, 40 S, 60 S, 80 S, and polysomes), its integrity, and identity monitored by agarose gel electrophoresis (middle panel) and used for northern blot analysis (lower panels). Full length (FL) tRNA^Pro^ and the tRNA^Pro^ 5ʹ half are indicated.

Previous deep sequencing of the small RNome in CHO-K1 cells identified differentially expressed miRNAs [17,18] and piRNAs [19]. Here we re-analysed the data for other small ncRNAs and indeed found indications for high expression of tRNA halves and shorter tdRs (**Fig. S1**). The expression and function of tRFs and halves were shown before to be associated with stress in several studies, therefore, CHO-K1 cells from different stress conditions (heat shock, cold shock, stationary phase growth, oxidative stress, and oxidative stress followed by recovery) were used for screening for tdRs via northern blot analysis. Since there are characterized examples of tRFs and tRNA halves directly binding to ribosomes and in this way regulating translation [13,15], northern blot screening was performed on total RNA and on RNA isolated from the crude ribosome pellets in parallel. As a result of this screen, the tRNA^Pro^ 5ʹ half was detected predominately in the ribosome-containing pellet, but under stationary growth conditions also in the total RNA fraction (Fig. 1A). The tRNA^Pro^ 5ʹ half was also apparent in other mammalian cell lines such as in HeLa, HEK, BON, NCI, and Hep3B cells (Fig. 1B). As observed in CHO cells, the tRNA^Pro^ half was constitutively expressed and did not show a clear change upon stress induction neither in HeLa nor in HEK cells (**Fig. S2**). Polysome profiling and consequent northern blot analysis confirmed the ribosome association of the tRNA^Pro^ half in CHO, HEK, and HeLa cells. In all three cell lines the tRNA^Pro^ half is present mainly in the 80S monosome fraction (HeLa, HEK) and in CHO and HEK cells also in the 60S subunit peak (Fig. 1C-E). The northern blot signal obtained in the small ribosomal subunit fractions (**e.g**. Fig. 1C) most certainly results from a spill-over from the light gradient fractions.

**Figure 2.**
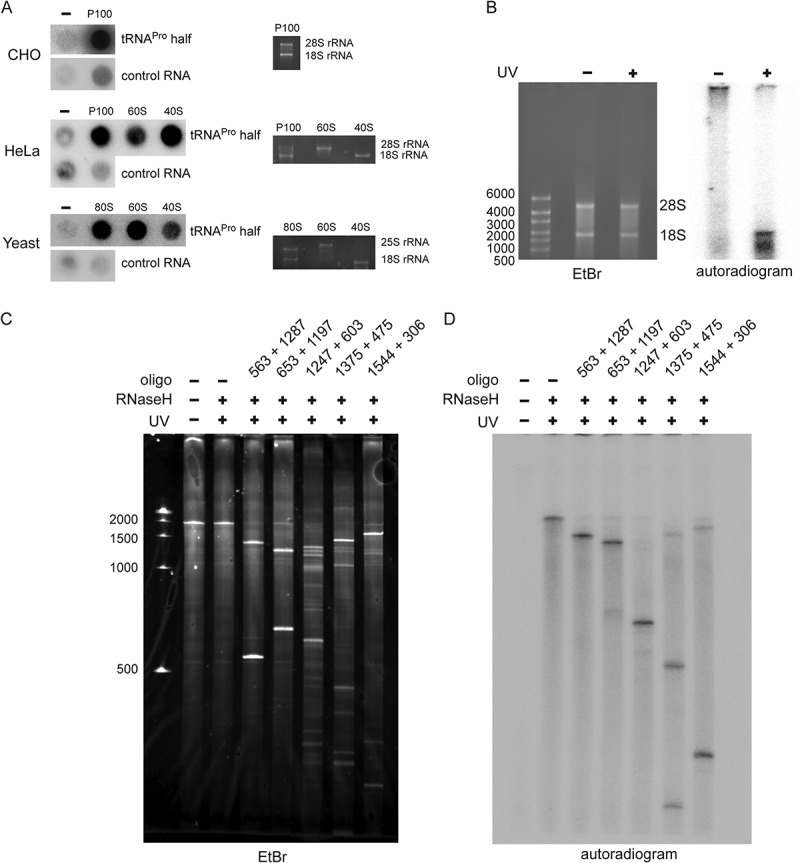
Binding of the tRNA^Pro^ half to ribosomes and ribosomal subunits *in vitro*. (A) Binding of the P^32^-labelled tRNA^Pro^ 5ʹ half to the crude ribosomal pellet (P100) and gradient-purified ribosomal subunits (40 S and 60 S) isolated from CHO cells, HeLa, and yeast cells was monitored by filter binding. Negative control (-) shows background signals in the absence of ribosomal particles. An unrelated small ncRNA (control RNA) from *H. volcanii* showed no (HeLa, yeast) or severely reduced (CHO) binding to ribosomal particles and serves as a binding specificity control. (B-C) Photocrosslinking of the tRNA^Pro^ half to human ribosomes. 5ʹ P^32^ – labelled tRNA^Pro^ half-carrying 4-thio-uracils was crosslinked to crude HeLa ribosomes at 366 nm followed by protease K treatment and RNA isolation. (B) After photo-crosslinking, RNAs were resolved on a 1% agarose gel. The ethidium bromide-stained gel and the autoradiogram thereof are shown. 18S rRNA and 28 S rRNA are labelled. (C) After photo-crosslinking, purified RNAs were subjected to RNase H treatment guided by DNA oligos complementary to different regions of 18S rRNA and the cleavage products were separated on 4% polyacrylamide denaturing gels. Ethidium bromide staining and autoradiogram are shown. Expected sizes of the produced rRNA fragments are indicated above each lane.

Binding of tRNA^Pro^ 5ʹ half to ribosomes and ribosomal subunits was confirmed by *in vitro* filter binding assays. P^32^-labelled tRNA^Pro^ half was shown to bind the crude ribosomal pellets (P100) isolated from CHO or HeLa cells (Fig. 2A). Filter binding assay using human or yeast ribosomal subunits showed binding to both 60 S and 40 S subunits, with slightly varying binding preferences (Fig. 2A). An unrelated short archaeal ncRNA was used as binding specificity control [20] which resulted in no or very diminished binding to mammalian ribosomes. The obtained binding data with the tRNA^Pro^ half are compatible with a binding site at or near the subunit interface. To test this assumption, UV crosslinking experiments employing a 5ʹ-^32^P labelled and 4-Thio-U-containing tRNA^Pro^ half was performed. The tRNA^Pro^ half crosslinked exclusively to 18S rRNA of HeLa 80S ribosomes (Fig. 2B). Using a similarly 5ʹ-^32^P labelled and 4-Thio-U-containing tRNA half from *T. brucei* [15] did not result in any detectable crosslink to rRNAs (**Fig. S3**) therefore highlighting the specificity of the observed effects. Employing a 3ʹ-biotinylated and 4-Thio-U-containing tRNA^Pro^ half in crosslinking and streptavidin pull-down experiments did not identify any ribosomal protein (**Table S1**), thus suggesting the ribosome binding site being primarily composed of rRNA. RNaseH-mediated cleavage guided by anti-sense DNA oligos complementary to different parts the 18S rRNA allowed us to narrow down the cross-linking site(s) to the last 306 nucleotides of the 3′ end of 18S rRNA (Fig. 2C, D). Highlighting this region in the context of the human 80S cryo-EM structure [21] showed its location at the subunit interface (**Fig. S4**). These data are therefore in line with the assumption that both subunits contribute to the binding site as suggested by the filter binding results (Fig. 2A).

### tRNA^Pro^ 5ʹ half inhibits translation and triggers a low molecular weight product

**Figure 3.**
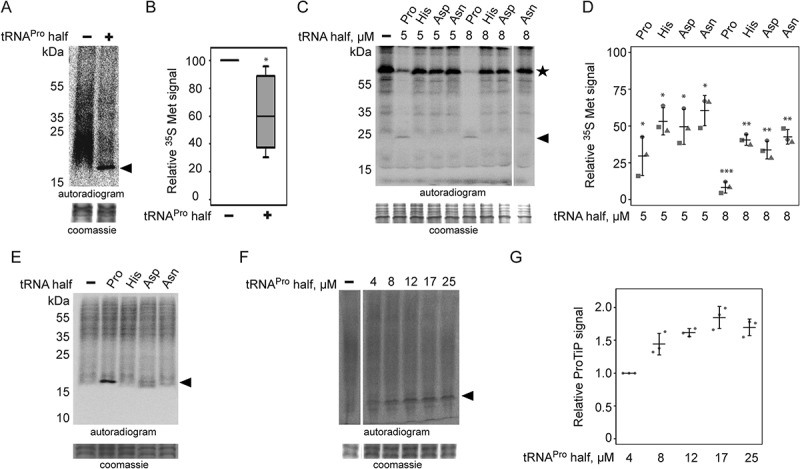
Effect of tRNA^Pro^ 5ʹ half on translation. (A) Autoradiogram of an SDS polyacrylamide gel after *in vitro* translation in the presence of the synthetic tRNA^Pro^ half using a CHO cell lysate. (B) Quantification of six *in vitro* translation experiments performed with CHO cell lysates in the absence (-) or presence (+) of the tRNA^Pro^ half. Values were normalized to the *in vitro* translation reaction with no tRNA^Pro^ half added. Box shows ± 25% of the mean, error bars show standard deviation. (C) Autoradiogram of an SDS polyacrylamide gel after *in vitro* translation of one reporter in the absence (-) presence of various synthetic tRNA 5ʹ halves (5 or 8 µM) using a rabbit reticulocyte cell lysate. In all cases, the tRNA 5ʹ halves are abbreviated by their respective amino acid three letter code. The asterisk marks the position of the full-length reporter protein. (D) Quantification of three *in vitro* translation experiments performed with rabbit reticulocyte cell lysate and one mRNA reporter in the presence of different tRNA 5ʹ halves (5 or 8 µM). Values were normalized to the *in vitro* translation reaction in the absence of the tRNA^Pro^ half. The horizontal lines indicate the mean and the error bars show standard deviations. (E) CHO *in vitro* translation in the presence of 8 µM synthetic tRNA halves. The apparent tRNA^Pro^ half-induced product (ProTiP) is indicated with a black arrowhead (here and in all panels). (F) CHO *in vitro* translation with increasing amounts of tRNA^Pro^ half added. (G) Quantification of ProTiP formation of three *in vitro* translation reactions as a function of increasing tRNA^Pro^ 5ʹ half concentration. The horizontal lines indicate the mean and the error bars show standard deviations. In (A), (C), (E), and (F) the coomassie stained protein bands of the lysate serve as a loading control. In (B) and (D) significant differences were determined using the 2-tailed paired Student’s t-test (***p < 0.001, **p < 0.01, *p < 0.05).

To test if the ribosome-associated tRNA^Pro^ half can influence protein synthesis, *in vitro* translation was performed based on a CHO cell lysate in the presence of the synthetic 35 nt long tRNA^Pro^ half. Addition of tRNA^Pro^ half reproducibly led to an inhibition of global protein biosynthesis to a varying extent (Figs. 3A, B). However, when using a rabbit reticulocyte-based translation system with a single mRNA reporter, the picture became clearer and very low levels of protein synthesis were reproducibly observed upon addition of the tRNA^Pro^ 5ʹ half (Fig. 3C). Addition of other tRNA 5ʹ halves had only comparably mild effects on translation (Figs. 3C, D). Addition of a DNA version of the tRNA^Pro^ half on the other hand showed no effect on protein biosynthesis (**Fig. S5**). Based on these experiments we decided to focus our subsequent research on the tRNA^Pro^ 5ʹ half.

During these experiments, we noticed that besides inhibition of translation, addition of the synthetic 5ʹ tRNA^Pro^ half into *in vitro* translation reactions led to the formation of a specific band that migrates like a ~ 17 kDa product on SDS-containing polyacrylamide gels (Figs. 3A, C). This proline tRNA half-induced product (to which we refer to as ProTiP) is specific to the tRNA^Pro^ 5ʹ half and is not produced in the presence of any other tested tRNA half (Fig. 3C, E). Formation of ProTiP was found to be dose-dependent as addition of increasing amounts of the tRNA^Pro^ 5ʹ half stimulates its production (Fig. 3F, G).

**Figure 4.**
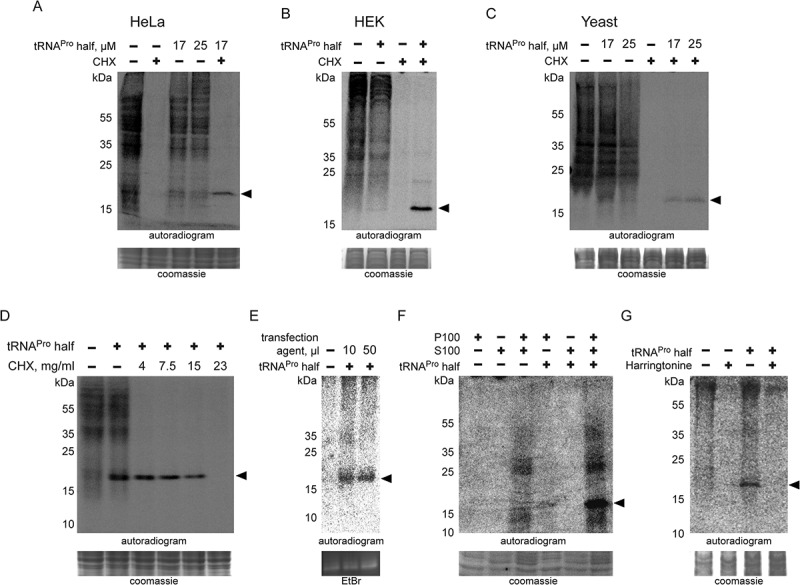
Effect of the tRNA^Pro^ half on translation in different eukaryotic systems. Autoradiograms of *in vitro* translation reactions performed using (A) HeLa, (B) HEK and (C) yeast extracts in the absence or presence of the tRNA^Pro^ 5ʹ half. Cycloheximide (CHX) with an f.c. of 7.5 mg/ml was used to inhibit global translation. ProTiP is indicated with a black arrowhead. (D) *In vitro* translation using CHO extracts and ProTiP formation (arrow head) was monitored in the presence of increasing amounts of cycloheximide (CHX). (E) Formation of ProTiP *in vivo*. 300 pmol of synthetic tRNA^Pro^ half was transfected into HeLa cells and the cells were subsequently incubated with S^35^ methionine. RNA isolated after metabolic labelling performed with (+) or without (-) transfected tRNA^Pro^ half was separated on a denaturing gel and visualized via autoradiography. The ethidium brome (EtBr) strained gel serves as a loading control. (F) Autoradiogram of *in vitro* translation reactions with HeLa ribosome-containing pellets (P100) or the corresponding ribosome-free supernatants (S100). Newly synthesized proteins, as well as ProTiP (black arrowhead), were produced only once P100 and S100 were combined. 100 pmol of tRNA^Pro^ half was used. (G) Inhibition of global translation in CHO extracts and ProTiP formation in the absence (-) or presence (+) of harringtonine (f.c. 2.5 mg/ml). In (A), (B), (C), (D), (F), and (G) the coomassie stained protein gels serve as loading controls.

Alignment of different tRNA^Pro^ genes shows high sequence identities in its 5ʹ region among several species (**Fig. S6**) suggesting a possible conservation of the tRNA^Pro^ 5ʹ half function. To test the effect of this tRNA half on translation in other biological systems, the same tRNA^Pro^ half was added to *in vitro* translation systems based on HeLa, HEK, or yeast cell extracts. Similar to the CHO and rabbit reticulocytes systems, addition of the tRNA^Pro^ 5ʹ half led to global translation inhibition and accumulation of ProTiP in all tested systems (Fig. 4A-C). We noticed that the addition of the tRNA^Pro^ half in the presence of low concentrations of the translation elongation inhibitor cycloheximide did not inhibit but even enhanced ProTiP formation. This increased ProTiP abundance, however, does not result from a putative stimulatory effect of low cycloheximide concentrations on tRNA^Pro^ half ribosome binding (**Fig. S7**). In cycloheximide titration experiments it turned out that only under higher concentrations of the inhibitor also ProTiP formation was reduced (Fig. 4D). The fact that tRNA^Pro^ half-mediated translation inhibition and ProTiP formation were observed in mammalian and yeast cell extracts suggests that the ribosome-binding site and/or the mode of action is conserved between different eukaryal translation systems. To test if ProTiP formation represents a peculiarity of *in vitro* translation systems or is also produced in living cells, the tRNA^Pro^ half was transfected into HeLa cells. Metabolic labelling in the presence of ^35^S methionine after transfection led to the production of ProTiP *in vivo* (Fig. 4E). These data show that tRNA^Pro^ half-mediated ProTiP production is not solely an *in vitro* translation phenomenon but occurs in living mammalian cells as well.

### ProTiP formation requires genuine protein biosynthesis

Several of the above-shown experiments suggest that ProTiP formation is a translation-dependent event. To substantiate these observations, HeLa cell extracts were divided via 100,000 xg centrifugation into the crude ribosomal pellet (P100) and the post-ribosomal supernatant (S100). *In vitro* translation activity only occurred once P100 and S100 fractions were combined (Fig. 4F). Addition of the tRNA^Pro^ 5ʹ half to P100 or S100 separately did neither result in translation activity nor in ProTiP formation. However, upon combination of the P100 and S100 fractions protein synthesis occurred and caused the appearance of ProTiP in the presence of the tRNA^Pro^ half (Fig. 4F). This experiment demonstrates that ProTiP formation indeed requires genuine translation. Support for this conclusion came from the fact that ProTiP synthesis could be influenced by the translational inhibitor harringtonine, which immobilizes eukaryotic ribosomes immediately after the initiation of translation. Harringtonine completely abolished global translation and also the production of ProTiP in the presence of the tRNA^Pro^ half (Fig. 4G). As mentioned above, similar effects were observed upon addition of elevated concentration of the tRNA translocation inhibitor cycloheximide (Fig. 4D).

### The chemical nature of ProTiP

**Figure 5.**
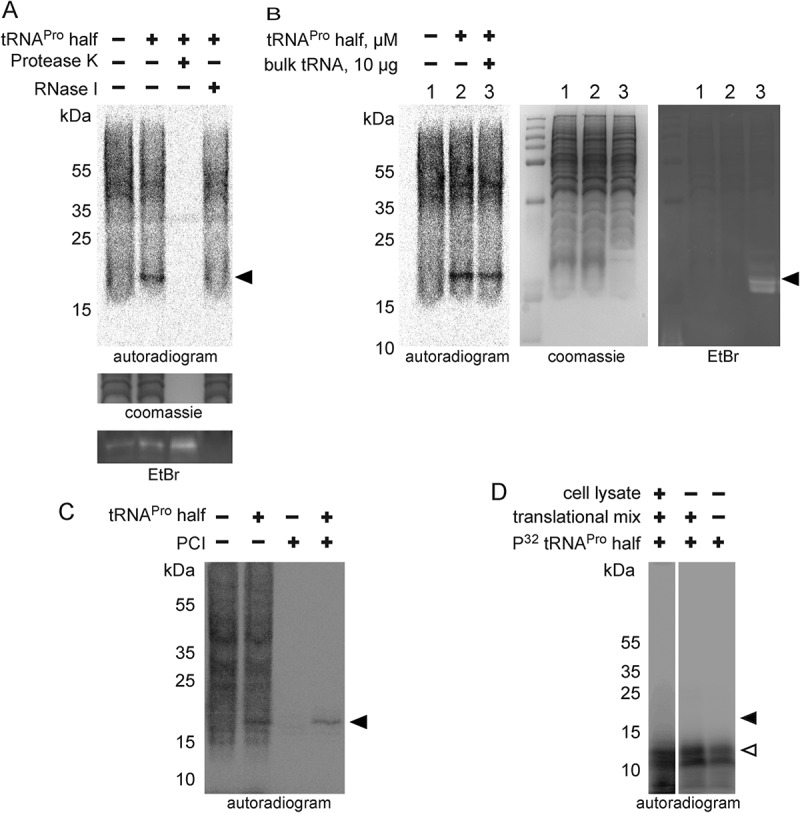
Chemical nature of ProTiP. (A) The products of *in vitro* translation using CHO cell lysates were incubated with proteinase K or RNase I. Protease K digests all newly translated and cell lysate proteins, as well as ProTiP (arrowhead). Also, RNAse I digestion removes ProTiP. The coomassie or ethidium bromide-stained gels serve as controls for efficient protein and RNA digestions, respectively. (B) Spike-in of yeast bulk tRNA (lane 3) after CHO *in vitro* translation in the absence (lane 1) or presence (lane 2) of 8 µM tRNA^Pro^ 5ʹ halves shows that ProTiP (arrowhead) migrates in the size range of tRNAs in the SDS polyacrylamide gel used. The gel was stained with coomassie (loading control) or with ethidium bromide (for visualization of the spiked-in tRNAs). (C) *In vitro* translation reactions (with or without tRNA^Pro^ halves) were loaded before (-) or after (+) phenol/chloroform/isoamyl alcohol (PCI) extraction on a denaturing SDS gel. ProTiP (black arrowhead) can be extracted into the water phase during PCI treatment. (D) Autoradiogram of an SDS polyacrylamide gel after *in vitro* translation in the presence of the internally ^32^P-labelled tRNA^Pro^ half (open arrowhead). Migration of the labelled tRNA^Pro^ half did not change after reactions in the presence of the cell lysate and the translational mix, thus demonstrating that ProTiP does not contain the tRNA^Pro^ 5ʹ half. Expected size of ProTiP is indicated with the black arrowhead.

ProTiP requires translation and it migrates as a ~ 17 kDa band on SDS gels thus indicating ProTiP of being a polypeptide. Indeed, *in vitro* translation followed by protease K digestion completely eliminated the ProTiP band on the gel (Fig. 5A). These findings are compatible with the view that addition of tRNA^Pro^ 5ʹ half into *in vitro* or *in vivo* translation reactions stimulates the production of one or several proteins that run as ~17 kDa products on denaturing gels. Ethidium bromide staining of the SDS gel showed that small RNAs, in particular tRNAs, also migrated in the same region of the gel as ProTiP (Fig. 5B). To test if ProTiP also contains RNA, *in vitro* translation reactions were subjected to an RNase I treatment. ProTiP was shown to be RNase sensitive since the band disappeared after nuclease digestion (Fig. 5A). The observation that ProTiP could be extracted into the water phase during phenol/chloroform extraction further indicates that ProTiP contains not only a peptidyl or protein part but also an RNA component (Fig. 5C).

**Figure 6.**
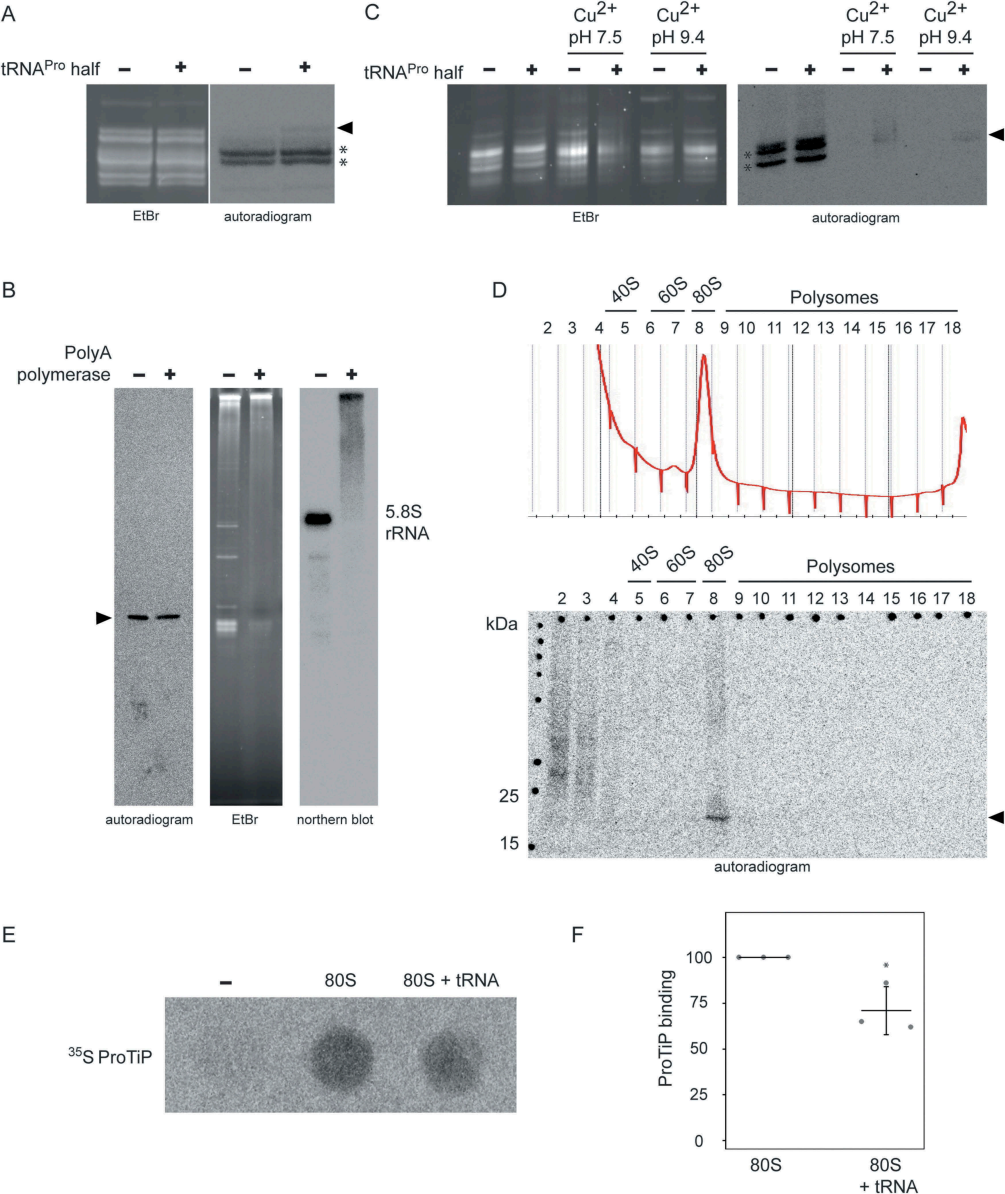
ProTiP is likely a peptidyl-tRNA. (A) Autoradiogram and ethidium bromide staining of an acidic gel after *in vitro* translation performed in the absence (-) or presence (+) of the tRNA^Pro^ 5ʹ half. Charged tRNA^iMet^ and tRNA^Met^ are labelled by asterisks. ProTiP is indicated with the black arrowhead. (B) Autoradiogram of an 8% denaturing polyacrylamide gel showing the position of ProTiP (arrowhead) before (-) and after (+) polyadenylation. Middle and left panels: Ethidium bromide staining and northern blot against 5.8S rRNA demonstrates the efficiency of polyadenylation. (C) Copper sulphate treatment of RNA isolated after *in vitro* translation performed in the presence (+) or absence (-) the tRNA^Pro^ half. The location of ProTiP, which was insensitive to copper sulphate treatment, is indicated in the autoradiogram with the black arrowhead. The Met-tRNA^Met^ molecules are labelled with asterisks. (D) Fractions of the polysome profile after *in vitro* translation in the presence of the tRNA^Pro^ half were subjected to isopropanol precipitation. Precipitated RNA was separated on a denaturing gel and visualized via phosphorimaging. ProTiP is indicated with the black arrowhead. (E) Binding of ProTiP to purified HeLa 80 S *in vitro* with and without pre-incubated bulk tRNAs. Ribosome-bound ^35^S-ProTiP was determined by filter binding. (F) Quantification of three individual binding experiments. Values were normalized to the binding of ProTiP to ribosomes without the addition of bulk tRNAs. The error bars show standard deviations (*p < 0.05).

To better reveal the chemical identity of ProTiP, we first focused on the characterization of its RNA part. By utilizing a 5ʹ-^32^P labelled (Fig. 5D) or a 3ʹ biotinylated tRNA^Pro^ half (**Fig. S8**) during *in vitro* translation we could clearly demonstrate that the tRNA^Pro^ 5ʹ half itself is not part of ProTiP. To better estimate the size of ProTiP, samples extracted via phenol/chloroform treatment after *in vitro* translation were loaded on an acidic gel. On acidic gels, ProTiP migrated in the range of the tRNA pool, slightly above methionyl-tRNAs (Fig. 6A). To characterize the 3ʹend of ProTiP, a polyadenylation experiment was designed. If the RNA moiety of ProTiP has a free 3ʹ end, it will be susceptible to polyadenylation and consequently would migrate more slowly on polyacrylamide gels. In contrast, if the RNA 3ʹ end is protected it would be insensitive to polyadenylation and, thus, would not change its electrophoretic mobility. Polyadenylation performed on RNA extracted after *in vitro* translation in the presence of tRNA^Pro^ half lead to an upshift of the majority of RNA on ethidium bromide-stained gels, while the autoradiogram of the same gel showed that ProTiP migration remained unchanged upon polyadenylation (Fig. 6B). As a control, northern blot analysis was performed on the same gel using a probe against the 5.8S rRNA. After polyadenylation, the 5.8 S rRNA signal was quantitatively upshifted on the gel demonstrating efficient poly(A) addition (Fig. 6B). Therefore, the insensitivity of ProTiP to polyadenylation demonstrates that it does not contain a free 3ʹ OH end. Electrophoretic mobility of ProTiP within the size range of tRNAs on acidic gels and the insensitivity to polyadenylation suggests that ProTiP could be a tRNA with one or several amino acids attached to its 3ʹ end thus resembling aminoacyl – or peptidyl-tRNAs, respectively.

To distinguish if ProTiP is an aminoacyl-tRNA or a peptidyl-tRNA, copper sulphate treatment was performed. Copper sulphate selectively deacylates aminoacyl-tRNAs with a free N-terminus but leaves peptidyl-tRNA intact [22]. This method is based on the ability of heavy metal ions to form stable complexes with α-amino acid esters and to promote hydrolysis of the ester bond, while any substituent on the α-carbon of the amino acid ester decreases the rate of hydrolysis [23]. Copper sulphate treatment on RNA extracted after *in vitro* translation showed that ProTiP stays intact while the methionyl-tRNAs were effectively deacylated (Fig. 6C). Polysome profiling performed after *in vitro* translation reactions in the absence or presence of the tRNA^Pro^ 5ʹ half showed that addition of tRNA^Pro^ half leads to a slight but reproducible increase of the 80S monosome peak on polysome gradients (**Fig. S9**). To trace ProTiP within the density gradient, fractions of the polysome gradient were subjected to RNA precipitation and the samples subsequently loaded on a denaturing SDS gel. The autoradiogram of the dried gel reproducibly showed the association of ProTiP with the 80S monosome fraction (Fig. 6D). Cumulatively, the presented evidence suggests that ProTiP is a tRNA^Pro^ 5ʹ half-dependent translation product composed of both an RNA and a peptidyl part and is likely a peptidyl-tRNA. To substantiate this conclusion, two control experiments were performed. First, we subjected ProTiP to low concentrations of RNase T1 or RNase I and expected the radioactive signal to down shift in denaturing gels. Indeed, the full-length ProTiP band diminished with the concomitant appearance of specific shorter radioactive products as one would expect if ProTiP is a peptidyl-tRNA (**Fig. S10**). Second, we designed ribosome-binding competition experiments of ^35^S-ProTiP with deacylated tRNAs under P-site binding conditions. Indeed, the signal of ribosome-bound ProTiP reproducibly decreased upon tRNA addition indicating that they share an overlapping binding site on the 80S ribosome (Fig. 6E, F).

### Discussion

In this study, we screened for tRNA halves and tRNA fragments (tRFs) in various mammalian cell lines and uncovered a ribosome-associated tRNA^Pro^ 5ʹ half (Fig. 1C-E and **Fig. S2**). Ribosome association was confirmed *in vitro* via filter binding assays and photo-crosslinking experiments (Fig. 2). Addition of synthetic tRNA^Pro^ 5ʹ half to *in vitro* translation reactions showed unique effects on protein biosynthesis. First, the tRNA^Pro^ 5ʹ half inhibits global translation (Fig. 3A-D). Previously, it has been described that several stress-induced tRNA 5ʹ halves (5ʹ tiRNAs) inhibit translation in human cells by titrating away crucial translation initiation factors [24] and promote stress granule formation [25]. In contrast to the previously described tiRNAs, the tRNA^Pro^ 5ʹ half identified here does not contain the 5ʹ terminal oligoguanosine motif uncovered before on tiRNAs of being central for its inhibitory function and it also does not show stress-dependent expression (Fig. 1A). Moreover, while a DNA analogue of a 5ʹ tiRNA was sufficient to inhibit translation [26], a DNA version of the tRNA^Pro^ half failed to inhibit protein biosynthesis (**Fig. S5**). These findings also suggest that the interaction of the tRNA half with the rRNA target is not entirely depending on simple base pairing. Altogether, our data suggest that the tRNA^Pro^ 5ʹ half possesses a so far unknown mechanism of translation inhibition in mammalian cells. The second effect of the 5ʹ tRNA^Pro^ half on translation was the formation of a low molecular weight product that is bound to 80S monosomes (Fig. 6D) and migrates like a ~ 17 kDa product in SDS gels (Fig. 3A,C,E,F). The tRNA^Pro^ half–induced product (named ProTiP) was not only observed *in vitro* but also after the transfection of tRNA^Pro^ halves into HeLa cells (Fig. 4E). By performing streptavidin pull-down experiments employing a 3ʹ biotinylated tRNA^Pro^ half after *in vitro* translation **(Fig. S8**), or by utilizing a ^32^P-labelled tRNA^Pro^ half during the translation reaction (Fig. 5D), we could exclude the possibility that the tRNA^Pro^ half itself is part of ProTiP. Both, translation inhibition and ProTiP formation were observed in several eukaryotic translation systems therefore indicating a conserved function and ribosome-binding site of the tRNA^Pro^ 5ʹ half (Fig. 4A-C). Cumulative experimental evidence indicates that ProTiP is a hybrid molecule containing both an RNA and a peptidyl part (Fig. 5A, C and **Fig. S10**). Furthermore, ProTiP migrates in denaturing gels in the size range of tRNAs (Figs. 5B and 6A-C), associates with 80S monosomes in polysome profiles (Fig. 6D) and can be chased off the ribosome by tRNA addition (Fig. 6E,F). Taken together the obtained data are in line with the conclusion that ProTiP is a peptidyl-tRNA.

**Figure 7.**
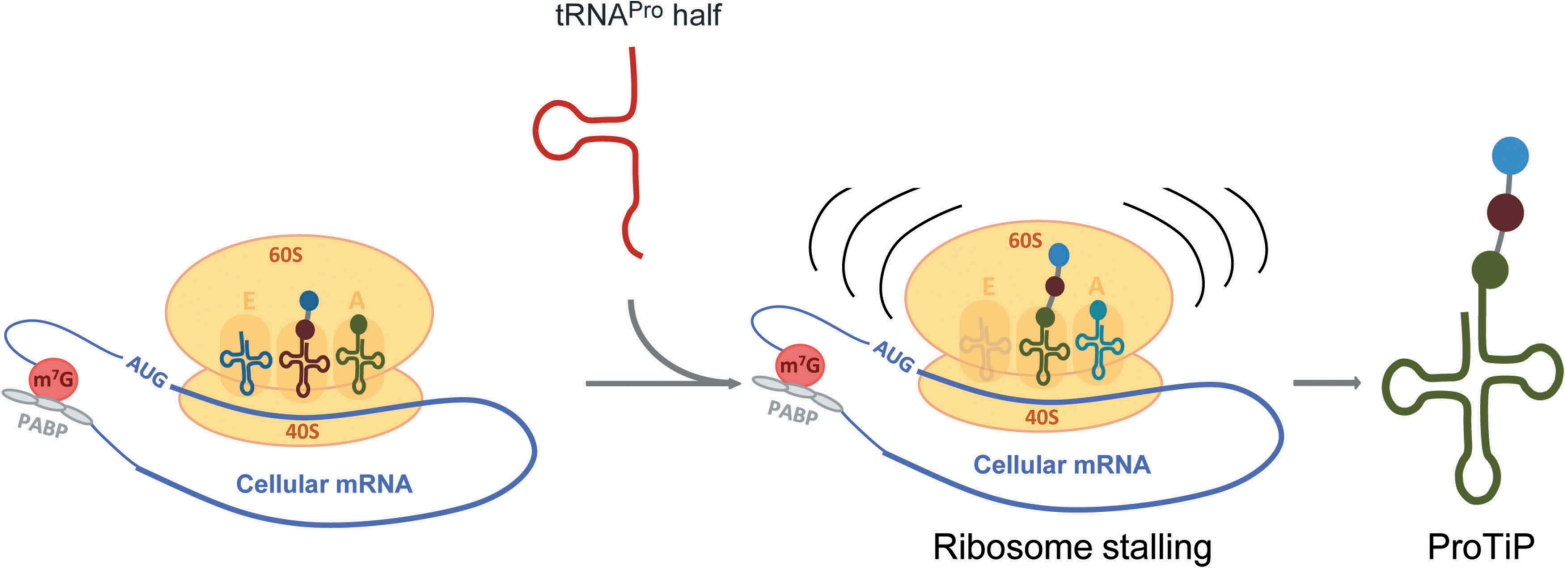
Model of the effect of the tRNA^Pro^ 5ʹ half on translation. The tRNA^Pro^ is processed into the tRNA^Pro^ 5ʹ half through cleavage at the anticodon region. The tRNA^Pro^ 5ʹ half binds to the ribosome and likely causes ribosome stalling. Consequently, translation is inhibited globally resulting in the accumulation of the peptidyl-tRNA inside the stalled ribosomes that we identified as ProTiP.

Previous studies have shown that under certain conditions distinct aminoacyl-tRNAs are modified at the N-terminus resulting in the block of translation [27,28]. In both cases, the produced modified aminoacyl-tRNAs were detected as a sharp clean band migrating on polyacrylamide gels in the region of the tRNA pool, in a similar fashion like ProTiP. However, if ProTiP was an N-terminally modified aminoacyl-tRNA, its production would not be translation dependent and ProTiP would migrate together with free tRNAs on polysome profiles after *in vitro* translation. The fact that after *in vitro* translation ProTiP stays associated with 80S monosomes, but was absent in the light density gradient fractions containing unbound RNAs (Fig. 6D) suggests that the accumulated product is a peptidyl-tRNA rather than N-modified aminoacyl-tRNA. Thus, our data are compatible with the scenario that binding of the tRNA^Pro^ 5ʹ half to ribosomes might lead to ribosome stalling, and consequently to translation inhibition followed by the accumulation of peptidyl-tRNA(s) (ProTiP) (Fig. 7). Despite the fact that we were unable to unequivocally identify the molecular nature of ProTiP by a variety of complementary methods, several conclusions can be drawn based on our findings. Since ProTiP always migrates as a sharp band on denaturing gels suggests that it contains one specific tRNA body but could still be heterogeneous in the length and composition of the peptidyl part. As ProTiP is produced in different translation systems on different mRNAs, it is likely that the formed peptidyl-tRNAs have heterogeneous amino acid sequences with a varying number of residues. Variation in the amino acid sequence and slight differences of the peptidyl length would not dramatically change the bulk charge of the peptidyl-tRNA, and consequently its migration on denaturing polyacrylamide gels. Therefore, it is reasonable to assume that ProTiP represents a group of peptidyl-tRNAs with one particular tRNA body and different short peptidyl chains. The ability to extract ProTiP during a phenol/chloroform treatment into the water phase indicates that the peptidyl part is probably shorter than 80 amino acids [29]. Since cycloheximide allows only one round of elongation after ribosome binding [30], the production of ProTiP in the presence of cycloheximide pre-incubated with ribosomes (see Material and Methods) suggests that the peptidyl part contains only a few amino acids.

Some of the previously identified tdRs, including short tRFs as well as tRNA half molecules, have been appreciated in the recent past of possessing biological roles in various model organisms and are therefore more than degradation intermediates of tRNA homoeostasis. Accumulating evidence suggests that tdRs are a functionally very heterogonous class of ribo-regulators [2,6,31]. A subclass of these tRNA fragments effectively associates with the ribosome and are therefore included into the growing class of ribosome-associated ncRNAs (rancRNAs) [9]. However, even those tdRs that target the ribosome turned out to influence protein production in a markedly different manner. While some affect translation globally in an inhibitory [12,24] or stimulatory [15] manner, the tRNA^Pro^ 5ʹ half reported here, reveals yet another aspect of tdR biology. While it is functionally analogous to previously identified tRNA 5ʹ halves [24] in the sense that it globally down regulates protein production, it is unique in triggering the formation of ProTiP, a small molecular weight product. Our attempts to characterize the molecular nature of ProTiP suggest that it is peptidyl-tRNA likely produced as a consequence of tRNA^Pro^ half-mediated ribosome stalling. Future work will have to reveal whether ProTiP is a mere byproduct of the ribosome arrest or if it possesses a physiological role by itself. While previously reported tRNA halves capable of inhibiting translation were generated by angiogenin specifically during certain stress conditions, called tiRNAs [24], the tRNA^Pro^ 5ʹ half reporter herein is produced in a stress-independent manner. Therefore, it is safe to suspect that the tRNA^Pro^ 5ʹ half is generated via a different biogenesis pathway and it might fulfil a cellular role distinct from tiRNAs in mammalian cells.

## Materials and methods

### Cell cultivation and stress application

CHO-K1 cells were cultivated in CD CHO medium (*Gibco*) supplemented with 8 mM glutamine (*Gibco*), 1x PenStrep (*Gibco*), and 2 ml/l anticlumping agent (*Gibco*) at 37ºC, 5% CO_2_. CHO cells were harvested during exponential growth at approximately 48 h after seeding with a cell density of 0.2 × 10^6^ cells/ml. HEK cells were cultivated in DMEM medium (*Gibco*) supplemented with 10% FCS (*Thermo Scientific*), 4 mM Glutamine (*Gibco*), and 1 x PenStrep (*Gibco*). For HeLa cells RPMI (*Gibco)* medium supplemented with 10% FCS, 4 mM glutamine (*Gibco*) and 1 x PenStrep (*Gibco*) was used. For northern blot analysis, the following stress conditions were applied: (I) stationary growth phase: for CHO-K1 cells 7 days after seeding with a cell density of 0.2 × 10^6^ cells/ml, for HeLa and HEK cells growth to the overconfluent stage, (II) heat shock treatment: 42°C for 30 min, (III) oxidative stress: incubation with 2 mM H_2_O_2_ for 2 h, (IV) recovery from oxidative stress: 2 mM H_2_O_2_ for 2 h followed by 2 h recovery in fresh media, (V) nutritional deprivation by incubation the cells in PBS for 2 h.

### Northern blot analysis

For northern blotting, 10–30 μg of RNA was separated by denaturating polyacrylamide gels (7 M urea) and subsequently electro-blotted onto nylon membranes (Amersham Hybond N+, *GE Healthcare*) as described [13]. All membranes were hybridized to a ^32^P-5′-end-labelled DNA probe complementary to the 5ʹ part of tRNA^Pro^ AATCATACCCCTAGACCAACGAGCC.

### Bulk ribosome pelleting

To generate crude ribosomal pellets, cell lysates were loaded on 0.5 ml 1.1 M sucrose cushion in 30 mM Hepes pH 7.6, 150 mM KOAc, 3.9 mM Mg(OAc)_2_, 4 mM DTT, 1 mM PMSF and centrifuged for 2.5 h at 200,000 xg in a Sorval M120+ miniultracentrifuge equipped with an S140AT rotor (*Thermo Scientific*). Obtained ribosomal pellets were resuspended in 1 x resuspension buffer (30 mM HEPES-KOH, pH 7.6; 150 mM KOAc, 3.9 mM Mg(OAc)_2_; 4 mM DTT; 1 x Tm complete (*Roche*); RNase inhibitor (*Thermo Fisher* or *Promega*). To prepare the more concentrated S100 extracts used in HeLa *in vitro* translation experiments, the cell lysate was directly ultracentrifuged without a sucrose cushion.

### Polysome profiling

For polysome profiling, exponentially growing cells were treated with freshly prepared cycloheximide to an f. c. of 100 µg/ml for 10 min. Cells were washed with PBS containing 100 µg/ml cycloheximide and opened in 1 x resuspension buffer containing 100 µg/ml cycloheximide by passing through a 25 G needle 30 times. Cell lysates were layered onto a 10–50% (w/v) linear sucrose gradient prepared in 30 mM HEPES-KOH, pH 7.6; 150 mM KOAc; 5 mM Mg(OAc)_2_, 4 mM DTT; 1 mM PMSF; 100 µg/ml cycloheximide. Samples were centrifuged at 39 krpm for 2 h 45 min at 4°C using an SW-41 swing-out rotor (*Beckman*). The gradient was pumped out from the top to the bottom using a Density Gradient Fractionation System (*Brandel*). Polysome proﬁles were generated by continuous measurement of the absorbance at 254 nm.

### Ribosome filter binding

In order to investigate the binding of the tRNA^Pro^ 5ʹ half to ribosomes and ribosomal subunits, the tRNA^Pro^ half was radioactively labelled at the 5ʹend with P^32^ – ATP or at the 3ʹ end with P^32^–pCp. For the binding reaction, 5 pmol of 80S/60S/40S was incubated with P^32^ tRNA^Pro^ half in 1 x binding buffer (30 mM HEPES-KOH, pH 7.6, 150 mM KOAc, 3.9 mM Mg(OAc)_2_) for 30 min at 35°C for CHO ribosomes and at 30°C for yeast ribosomes/ribosomal subunits. The reaction was diluted by the addition of 175 μl 1 x binding buffer. Then, samples were filtrated as described before [12]. As negative binding control an archaeal ncRNA was used (5ʹ-CAGAGUAACCCCUCGGACGCAUCCACAUGACCGAACUCAUCCCCCCUUCCCCCCUUCCCCAUUAGGUUCGGUCACCCCUUUA-3) (ref. 20).

In order to characterize the binding site of ProTiP on HeLa ribosomes by binding competition experiments with deacylated tRNA, ProTiP was first produced in *in vitro* translation reaction in the presence of the tRNA^Pro^ half in 20 replicates (see details below). To deplete endogenous tRNAs from the ribosome pool and to remove unincorporated S^35^ methionine, all reactions were pooled and subjected to ultracentrifugation for 1 h at 200,000 xg in a Sorval M120+ mini-ultracentrifuge equipped with an S140AT rotor (*Thermo Scientific*). To deacylate tRNA^Met^, the obtained pellet was resuspended in 2 x Laemmli buffer and boiled for 5 min at 95°C. Then, RNA was extracted using phenol/chloroform and the RNA pellet was resuspended in 70 μl water. For the binding competition reaction, 5 pmol of sucrose gradient – purified HeLa 80 S ribosomes in 1 x resuspension buffer in a volume of 18 μl was pre-incubated with 1 μl water or 1 μl of 40 μM yeast bulk tRNAs for 10 min at 35°C and 450 rpm. After pre-incubation, 5 μl of ProTiP-containing RNA was added to each sample and the reactions were incubated for another 10 min at 35°C and 450 rpm. Subsequently, the reactions were diluted by the addition of 175 μl 1 x binding buffer. Finally, the samples were filtered as described before [12].

### Crosslinking of 4–Thio-U-tRNA^Pro^ half to HeLa ribosomes

For the crosslinking experiments, 5–10 pmol of 5ʹ P^32^ labelled synthetic tRNA^Pro^ half carrying three photo-reactive 4-thiouridine residues at positions 4, 13, and 27 was incubated with 10 pmol of HeLa ribosomal pellet in 1 x resuspension buffer in a final volume of 20 μl for 15 min at 35°C and 450 rpm. Crosslinking was performed on ice at 366 nm for 10 min as described before [12]. After crosslinking, samples were subjected first to protease K treatment (f.c. 0.5 mg/ml of protease K, incubation for 30 min at 37°C), and then to phenol/chloroform extraction RNA extraction. Obtained RNA was resuspended in 20 μl of water. To test if the ^32^P-labled tRNA^Pro^ half was crosslinked to the rRNAs, 3 μl of RNA was separated on 1% agarose gel. After staining with ethidium bromide, the agarose gel was vacuum-dried at 70°C for 2 h and exposed to a phosphor imaging screen overnight. To narrow down the crosslinking site(s) of tRNA^Pro^ half on the 18 S rRNA, RNase H cleavage experiments were performed with DNA oligos annealing to different regions of 18S rRNA (18 S_563-582: AAAGGATTTAAAGTGGACTC, 18S_653-672: TTTTTAACTGCAGCAACTTT, 18 S_1247-1266: GTGAGGTTTCCCGTGTTGAG, 18S_1375-1394: CCAGAGTCTCGTTCGTTATC, 18S_1544-1563: CGTAGGGTAGGCACACGCTG). Prior to RNase H treatment, the extracted crosslinked RNAs were denatured at 70°C for 2 min. Each reaction contained 3 μl of denatured RNA, 10 pmol DNA oligo, 1x RNase H buffer (*New England Biolabs*), RNase inhibitor RNasine (*Promega*), and 3 U of RNase H. Cleavage was performed at 37°C for 1 hour. Cleavage products were analysed on 4% polyacrylamide gels containing 7 M urea. After staining with ethidium bromide, gels were vacuum-dried at 70°C for 2 h and exposed to phosphorimager screens.

### *In vitro* translation

Harvested cells were resuspended in equal volume of 1 x resuspension buffer. Cells were opened by passing through a 25 G needle 30 times. Cell debris was removed by centrifugation at 20,000 xg for 15 min at 4°C. To deplete endogenous methionine, the cell lysate was passed through a G25 sephadex spin column prepared in 30 mM HEPES pH 7.6, 150 mM KOAc, 3.9 mM Mg(OAc)_2_. Every *in vitro* translation reaction at the pre-incubation step had a total volume of 9.9 μl and contained 6–7 μl of cell lysate and 2.9–3.9 μl of water. For translation inhibition by antibiotics, cycloheximide, or harringtonine was dissolved in abs. ethanol and added first to the tubes and the solvent was evaporated. To test the effect of tRNA 5ʹ halves on translation 100–200 pmol of synthetic RNAs were added to the reactions (tRNA^Pro^ 5ʹ half GGCUCGUUGGUCUAGGGGUAUGAUUCUCGCUUAGG, tRNA^Asp^ 5ʹ half UCCUCGUUAGUAUAGUGGUGAGUAUCCCCGCCUG, tRNA^His^ 5ʹ half GCCGUGAUCGUAUAGUGGUUAGUACUCUGCGUUG, tRNA^Asn^ 5ʹ half GUCUCUGUGGCGCAAUCGGUUAGCGCGUUCGGCUG). Reactions were pre-incubated at 35°C at 450 rpm for 10 min. After pre-incubation, 2.1 μl of freshly prepared translational mix consisting of 1.2 µl 10 x translation cocktail (150 mM HEPES-KOH, pH 7.6, 750 mM KOAc, 19.5 mM Mg(OAc)_2_, 4 mM GTP, 17.5 mM ATP and 500 µM each of all 19 amino acids except methionine), 0.17 µl water, 0.08 µl 3 M creatine phosphate, 0.06 µl 20 mg/ml creatine phosphokinase and 0.625 µl S^35^-Methionine was added to every pre-incubated mixture increasing a total volume of reaction to 12 μl. *In vitro* translation was performed at 35°C at 450 rpm for 30 min. Reactions were stopped by the addition of 4 µl of 4 x Laemmli buffer and boiling at 95°C for 5 min.

For the translation of one reporter mRNA, Xef1 mRNA (*Thermo Scientific*) and rabbit reticulocyte cell lysate (*Thermo Scientific or Promega*) were used according to the manufactory’s protocol. To test the effect of tRNA halves on translation 50–150 pmol of synthetic RNAs were used. *In vitro* translation was stopped by the addition of Laemmli buffer and heating at 95°C for 5 min.

Samples were separated on 10-14% SDS-PAGE, stained with Coomassie Brilliant Blue and destained (several hours to overnight). For RNA visualization, gels were stained by soaking in a 1 x TBE solution containing 0.5 µg/ml ethidium bromide for 5–10 min. Afterwards, the gel was vacuum-dried at 70°C for 1 h and exposed to phosphor imaging screen overnight.

### Metabolic labelling

For metabolic labelling experiments, Hela cells were grown in 48-well plates to around 90% confluence. To transfect RNA into HeLa cells, 100 µl of FBS free Opti-MEM medium (*Gibco*) were combined with 300 pmol of the tRNA^Pro^ half, 10 µl or 50 µl of Lipofectamine RNAi MAX (*Invitrogen*) and incubated for 5 min at room temperature. Then, the mixture was added to one well and the cells were kept at 37°C and 5% CO_2_ for 5 min. To start the metabolic labelling 1 µl of S^35^ methionine (10 μCi/μl, *Hartmann Analytic*) was added to each well and the cells were incubated for another 30 min. Afterwards, the RNA was isolated with TRI reagent and separated on 12% SDS PAGE. To check the efficiency of RNA isolation and equal loading, the gel was stained with 1 x TBE containing 0.5 µg/ml ethidium bromide for 5–10 min. The gel was vacuum-dried at 70°C and exposed for 1 week to a phosphor imager screen to visualize ProTiP.

### Acidic gel electrophoresis

To separate charged tRNAs, RNA were separated on freshly prepared 15% denaturing urea acidic polyacrylamide gels (8 M Urea, 50 mM CH_3_ COONa pH 5.5). For the loading one volume of the RNA sample was combined with one volume of loading dye (93% v/v formamide, 150 mM NaOAc, pH 5.5; 10 mM EDTA, bromphenol blue, xylene cyanol). Subsequently, the gel was stained with 1 x TBE containing 0.5 µg/ml ethidium bromide for 10–15 min. The gel was vacuum-dried at 70°C and exposed overnight to a phosphorimaging screen.

### Polyadenylation

To characterize the 3ʹ end of ProTiP, total RNA was extracted from *in vitro* translation reactions performed in the presence of tRNA^Pro^ 5ʹ half and subjected to polyadenylation. Each reaction (total volume of 20 µl) contained 2 µl of 10 x Poly(A)Pol buffer (*New England Biolabs*), 1 µl RNA template (corresponding to 0.2 x of one *in vitro* translation reaction), 4 µl of 10 mM ATP, 1 µl RNasine (*Promega*), and 2 µl of Poly(A) Polymerase (*New England Biolabs*). Polyadenylation was carried out for 30 min at 37⁰C in a water bath. The reaction was stopped by addition of 22 µl of 2 x RNA loading dye. Afterwards, samples were separated on denaturing 8% polyacrylamide 7 M Urea 1 x TBE gel. After RNA separation, the gel was stained with 1 x TBE containing 0.5 µg/ml ethidium bromide for 10–15 min and then dried for 3 h at 70 ⁰C under vacuum. To detect the S^35^ signal of ProTiP, the dried gel was exposed to a phosphor imaging screen overnight. After the screen was scanned, the dried gel was rehydrated by incubation in 1 x TBE containing 0.5 µg/ml ethidium bromide. Once the gel was rehydrated, the quality of the RNA in the gel was checked under the UV light. To control for correct polyadenylation, the gel was blotted onto a nylon membrane and subsequently probed against 5.8S rRNA TCCTGCAATTCACATTAATTCTCGAGCTAGC by northern blot analysis.

### Copper sulphate treatment

Copper sulphate treatment was performed using a protocol developed previously [22] on RNA isolated after *in vitro* translation with CHO cell lysate. Each reaction in a total volume of 30 µl contained 40 mM CuSO_4_ x 5 H_2_O, 40 mM Tris, pH 7.5, or pH 8.9 and RNA isolated from 3 *in vitro* translation reactions. Reactions were incubated at 37°C for 1 h and afterwards subjected to RNA extraction. After PCI extraction, 1/10 of the obtained RNA was loaded on a 15% acidic gel.

## Supplementary Material

Supplemental Material

## Supplementary Material

The supplementary data for this article can be accessed here.
