# Supplementary material for: Modulation of mammalian translation by a ribosome-associated tRNA half
Source: RNA Biol. 2020 Mar 30;17(8):1125–36. doi: 10.1080/15476286.2020.1744296 (PMC7549673; doi:10.1080/15476286.2020.1744296)
Supplement: Supplemental Material [file KRNB_A_1744296_SM6924.pdf]

Supplementary Data

Modulation of mammalian translation by a ribosome-associated tRNA half

Yulia Gonskikh[1,2], Matthias Gerstl[3], Martin Kos[4], Nicole Borth[3], Markus Schosserer[3], Johannes Grillari[3,5,6], and Norbert Polacek[1,*]

[1]Department of Chemistry and Biochemistry, University of Bern, Freiestrasse 3, 3012 Bern, Switzerland

[2]Graduate School for Cellular and Biomedical Sciences, University of Bern, Bern, Switzerland

[3]Department of Biotechnology, BOKU - University of Natural Resources and Life Sciences, Vienna, Muthgasse 18, 1190 Vienna, Austria

[4]Biochemistry Center, University of Heidelberg, Heidelberg, Germany

[5]Christian Doppler Laboratory on Biotechnology of Skin Aging, Muthgasse 18, 1190 Vienna, Austria

[6]Ludwig Boltzmann Institute for Experimental and Clinical Traumatology, Donaueschingenstr. 13, 1200 Vienna, Austria

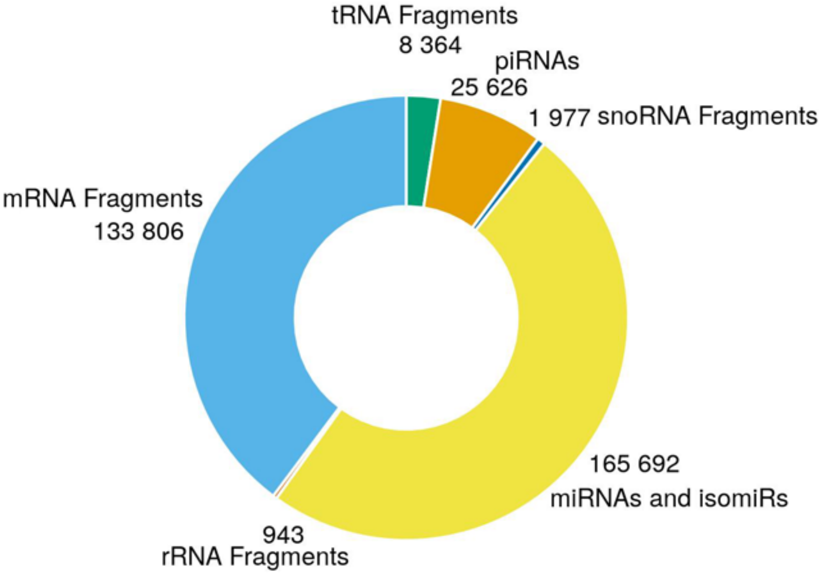

Supplementary Figure S1. The small RNome of CHO cells. Illumina sequencing of CHO small RNA libraries with a length of 18 to 36 nucleotides resulted in 4,703,659 unique reads. Out of these reads 165,692 sequences where annotated as miRNAs and isomiRs (yellow) (1). Furthermore, 133,806 mRNA fragments (sky blue), 25,626 piRNAs (orange), 8,364 tRNA fragments (green), 1,977 snoRNA fragments (dark blue), and 943, rRNA fragments (red) were identified (2). 4,367,251 sequences could not be annotated so far, since the *C. griseus* genome is still not finalized.

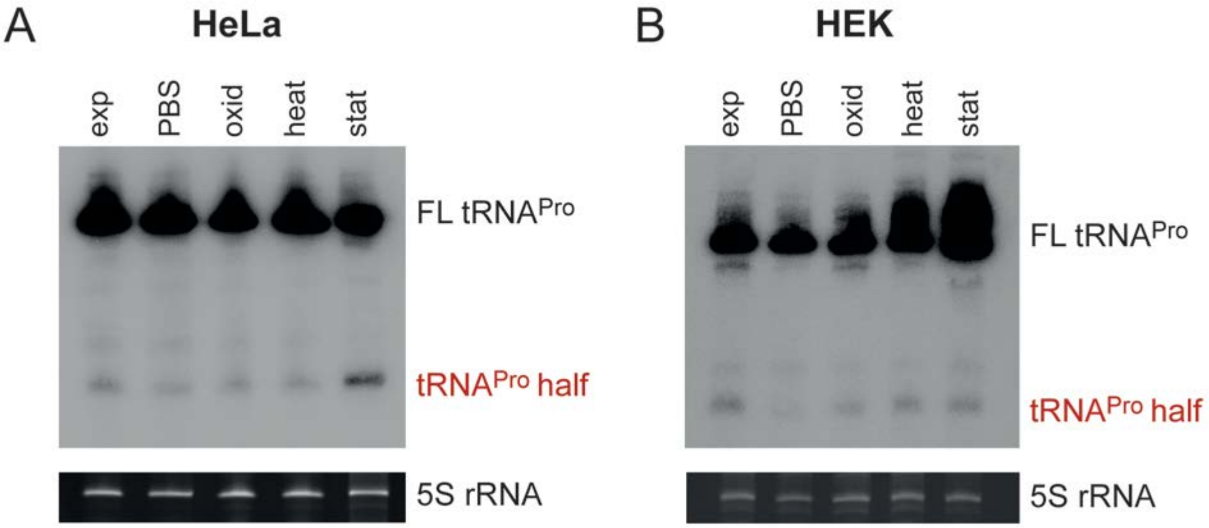

Supplementary Figure S2. Expression of the tRNA^{Pro} half. (A, B) Northern blot analysis of the 5’ tRNA^{Pro} half on 20 µg of total RNA isolated from unstressed and stressed HeLa and HEK cells, respectively. Applied stress conditions are indicated: nutritional stress (PBS), oxidative stress, oxidative stress with recovery, heat shock, and stationary growth phase. Full length (FL) tRNA^{Pro} and tRNA^{Pro} 5’ half are indicated. Ethidium bromide stained 5S rRNA serves as a loading control.

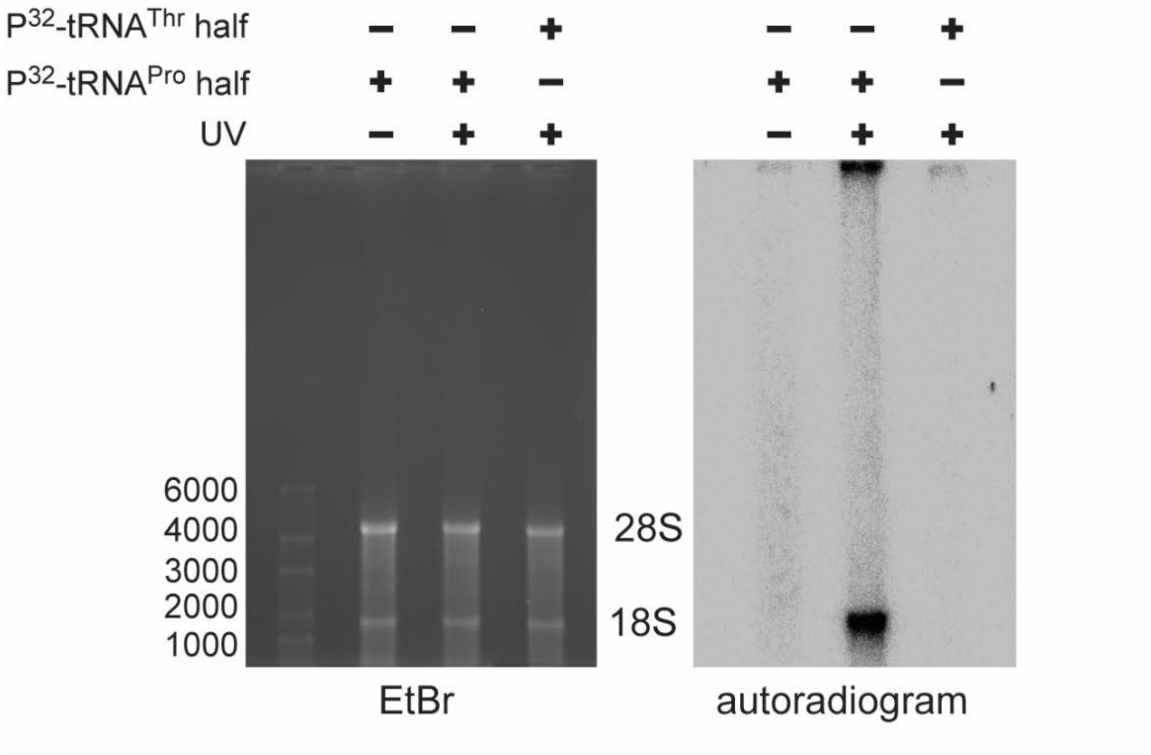

Supplementary Figure S3. Crosslinking of tRNA halves to HeLa ribosomes. 5' P^{32} –labeled human $tRNA^{Pro}$ 5' half or the *T. brucei* 5' ^{32}P–labeled $tRNA^{Thr}$ 3' half carrying 4-thio-uracils were crosslinked to crude HeLa ribosomes at 366 nm. After photo-crosslinking, RNAs were resolved on a 1% agarose gel. The ethidium bromide stained gel (left) and the autoradiogram of the same gel (right) are shown. Positions of 18S rRNA and 28S rRNA are indicated and an RNA size marker (in nt) is depicted on the left. Only the human $tRNA^{Pro}$ 5' half crosslinked to HeLa 18S rRNA.

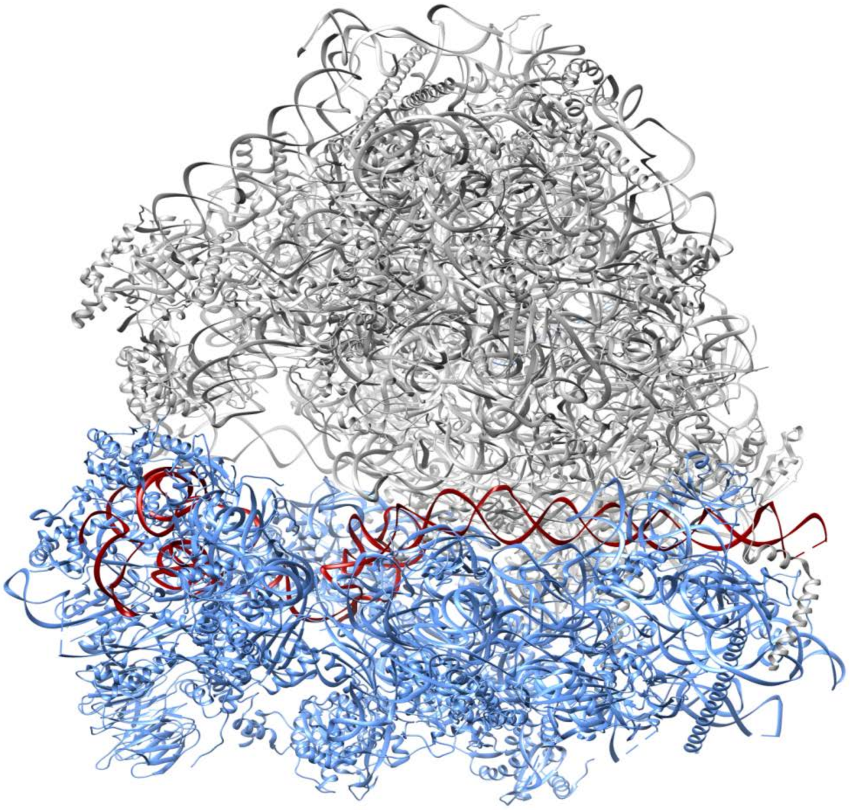

Supplementary Figure S4. Structure of the human ribosome. The $tRNA^{Pro}$ 5’ half crosslinks to a segment of 18S rRNA encompassing the last 306 nucleotides (red). The 60S subunit is grey and the 40S subunit is depicted in blue.

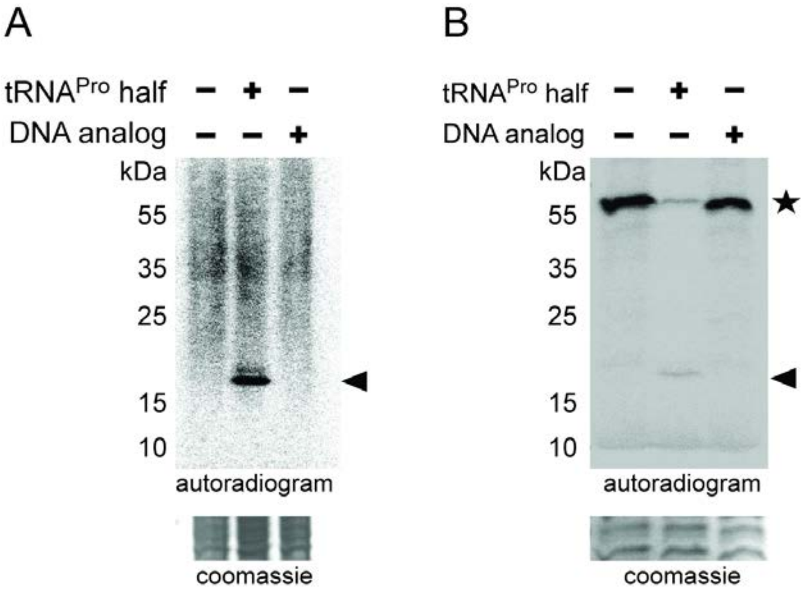

Supplementary Figure S5. *In vitro* translation in the presence of a DNA analog of the tRNA^{Pro} 5' half. Autoradiograms of SDS polyacrylamide gels after *in vitro* translation in the presence of 150 pmol of the tRNA^{Pro} 5' half or 150 pmol of its DNA analog using CHO (A) or rabbit reticulocyte (B) cell lysates. The full-length product of the single mRNA reporter in (B) is indicated with an asterisk. ProTiP is labeled with black arrowheads. Coomassie stained gels serve as a loading control.

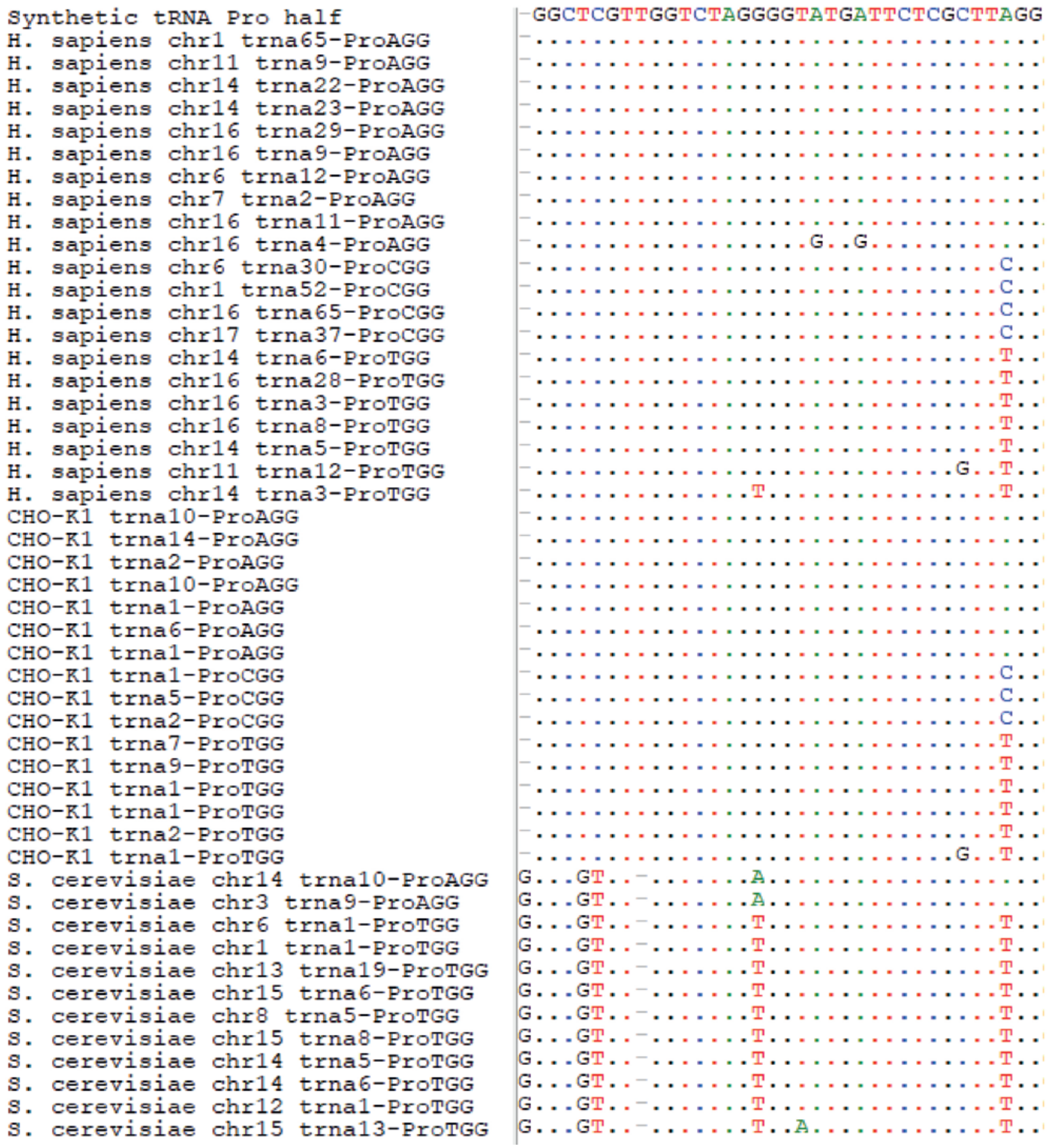

Supplementary Figure S6. Alignement of tRNA^{Pro} genes. Alignment of the tRNA^{Pro} 5' half to the 5'ends of tRNA^{Pro} genes from different organisms. tRNA^{Pro} is represented by 10 genes in the human genome, by 16 in CHO cells and 12 in yeast. Dots show sequence identities to the tRNA^{Pro} half (top line) whereas nucleotides indicated mismatches.

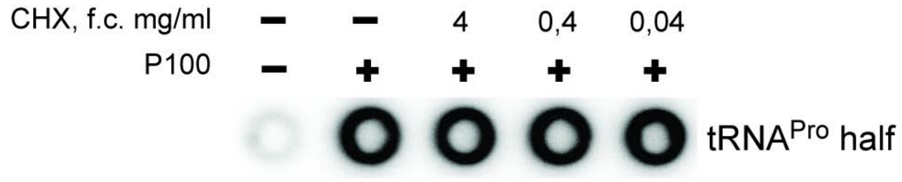

Supplementary Figure S7. Low concentrations of cycloheximide have no effect on tRNA^{Pro} half binding. The interaction of ^{32}P-labled tRNA^{Pro} half to crude CHO ribosomes (P100) was assessed by filter binding in the absence (-) or in the presence varying concentrations of the translation inhibitor cycloheximide (CHX). Cycloheximide binds to the 60S E-site and occupies a site that would clash with the very 3'-end of E-tRNA (3). Therefore tRNA^{Pro} 5' half and CHX do not seem to occupy overlapping binding sites on the mammalian ribosome.

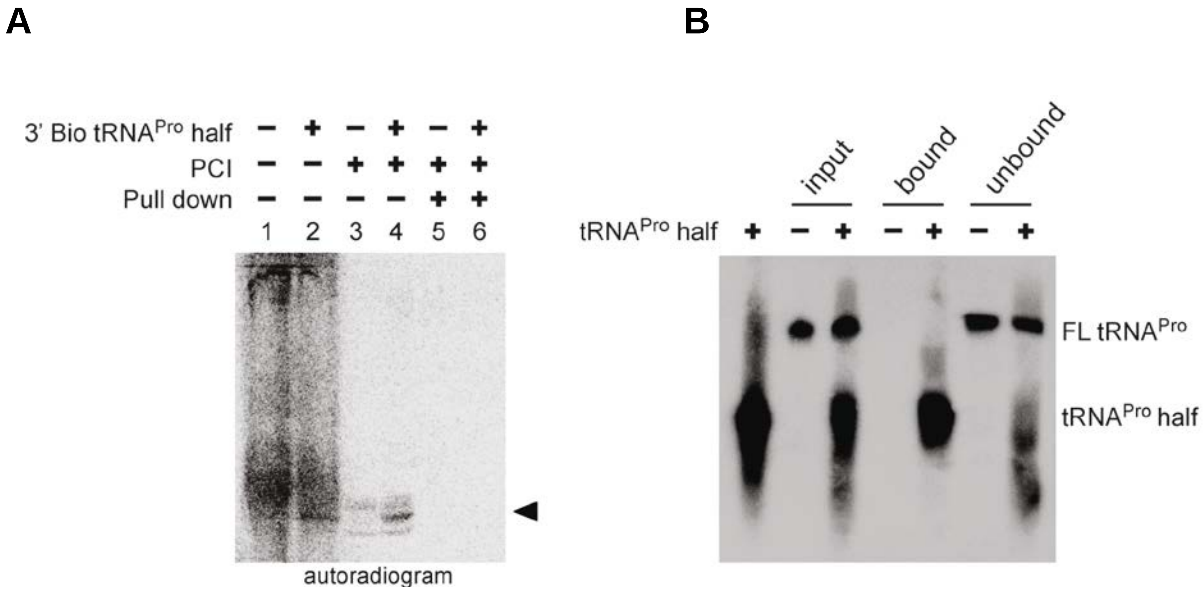

Supplementary Figure S8. tRNA^{Pro} half is not part of ProTiP. (A) Autoradiogram of an SDS polyacrylamide gel separating products of *in vitro* translation reactions performed in the absence (lane 1) or presence (lane 2) of the 3' biotinylated tRNA^{Pro} half. Reactions were also subjected to PCI RNA extraction (lanes 3 and 4). The 3' biotinylated tRNA^{Pro} half was purified on magnetic streptavidin beads from the PCI extracted RNA samples (lanes 5 and 6). ProTiP (arrow head) was not co-purified with the 3' biotinylated tRNA^{Pro} half (lane 6). (B) To confirm the efficiency of the carried out pull-down, northern blot against the tRNA^{Pro} half was performed on the same samples as in (A) (input samples, bound and unbound fractions). The strong signal of tRNA^{Pro} half in the input fraction, its depletion in the unbound fraction, and the absence of full length tRNA^{Pro} in the bound fraction demonstrates the efficient and specific tRNA^{Pro} half pull-down. Full length (FL) tRNA^{Pro} and the tRNA^{Pro} 5' half are indicated.

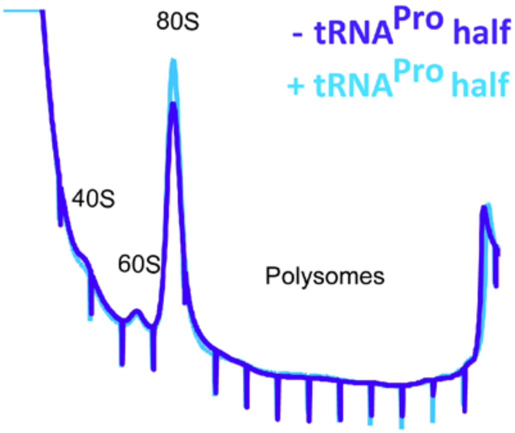

Supplementary Figure S9. ProTiP is likely a peptidyl-tRNA. Overlaid polysome profiles after *in vitro* translation reactions performed with and without the $tRNA^{Pro}$ 5' half. Profiles were normalized to the same square area under the entire profile

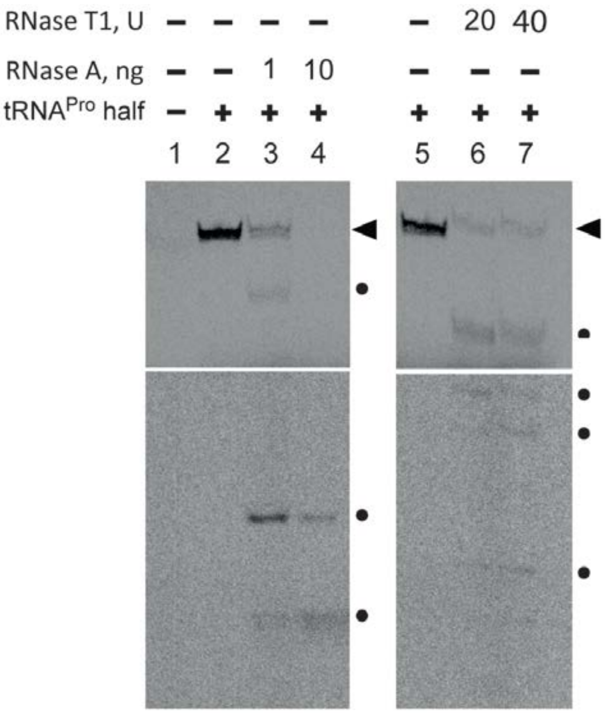

Supplementary Figure S10. RNase digestion of ProTiP. RNA extracted after *in vitro* translation using a CHO cell lysate in the absence (lane 1) or in the presence of $tRNA^{Pro}$ half (lanes 2-7) were subjected to RNase A or RNase T1 treatment, respectively. Products of the RNase digestion were separated on 12% polyacrylamide sequencing gel. Autoradiogram of the sequencing gel is shown. ProTiP is labeled with black arrowheads. Truncated products of the RNase digestions are indicated with black dots.

Supplementary Table S1

RNP analysis of the $tRNA^{Pro}$ 5' half. Proteins detected by mass–spectrometry to be significantly enriched after pull-down of 3'biotinylated 4-thio-U-$tRNA^{Pro}$ half from crude ribosomes after crosslinking at 366 nm. An analogous pull-down with the 3' biotinylated $tRNA^{Pro}$ half without 4-thio-U modification was used as a negative control. Adjusted p-values were calculated using the t-test.

Protein name		Fold change (4ThioU $tRNA^{Pro}$ half/ $tRNA^{Pro}$ half)	p Value
Q96AE4|FUBP1	Far upstream element-binding protein 1	7,17	3E-08
P68133|ACTS	Actin, alpha skeletal muscle	6,22	0,008
Q15056-2|IF4H	Eukaryotic translation initiation factor	5,26	5E-10
P16989|YBOX3	Y-box-binding protein	3,90	4E-04
P17655-2|CAN2	Isoform 2 of Calpain-2 catalytic subunit	3,54	4E-04
Q9UBF2-2|COPG2	Isoform 2 of Coatomer subunit gamma-2	3,47	2E-06
Q10471|GALT2	Polypeptide N-acetylgalactosaminyltransferase; Isoform 2	3,43	9E-06
Q12907|LMAN2	Vesicular integral-membrane protein	3,38	1E-07
Q04446|GLGB	1,4-alpha-glucan-branching enzyme	3,26	2E-08
P35237|SPB6	Serpin B6	3,26	1E-07
Q92945|FUBP2	Far upstream element-binding protein 2	3,14	2E-06
Q12933-4|TRAF2	TNF receptor-associated factor 2	3,04	9E-04
P52888|THOP1	Thimet oligopeptidase	2,99	8E-05
P24928|RPB1	DNA-directed RNA polymerase II subunit	2,98	2E-05
Q9Y2T2|AP3M1	AP-3 complex subunit mu-1	2,95	1E-03
P61204-2|ARF3	ADP-ribosylation factor 1	2,95	9E-08
Q32P44|EMAL3	Echinoderm microtubule-associated protein-like 3	2,90	4E-04
P40937-2|RFC5	Replication factor C subunit 5	2,90	4E-04
Q5VTR2|BRE1A	E3 ubiquitin-protein ligase	2,77	4E-04
P42704|LPPRC	Leucine-rich PPR motif-containing protein, mitochondrial	2,67	6E-05
P49419-2|AL7A1	Alpha-aminoadipic semialdehyde dehydrogenase	2,63	1E-04
P16083|NQO2	Ribosyldihydronicotinamide dehydrogenase	2,62	0,002
Q92734-4|TFG	Protein TFG	2,60	0,001
Q9UKS6|PACN3	Protein kinase C and casein kinase substrate in neurons protein 3	2,44	8E-04
Q13813|SPTN1	Spectrin alpha chain, non-erythrocytic	2,31	0,002
Q92890-3|UFD1	Ubiquitin recognition factor in ER-associated degradation protein 1	2,31	1E-05
P12270|TPR	Nucleoprotein TPR	2,17	2E-04
Q08170|SRSF4	Serine/arginine-rich splicing factor 4	2,12	6E-05
Q86TI2-4|DPP9	Dipeptidyl peptidase 9	2,11	7E-04
P13861-2|KAP2	cAMP-dependent protein kinase type II-alpha regulatory subunit	2,08	2E-04
P10398|ARAF	Serine/threonine-protein kinase	2,07	0,007
Q9Y2T3-2|GUAD	Guanine deaminase	2,07	6E-05
Q14847|LASP1	LIM and SH3 domain protein	2,07	0,003
Q06323-3|PSME1	Proteasome activator complex subunit 1	2,02	0,001
P40692-2|MLH1	DNA mismatch repair protein Mlh1	2,00	9E-04
Q9UPU5|UBP24	Ubiquitin carboxyl-terminal hydrolase 24	1,99	5E-05
Q9UPN3-4|MACF1	Microtubule-actin cross-linking factor 1	1,99	4E-04
Q9Y4L1|HYOU1	Hypoxia up-regulated protein 1	1,97	1E-04

O43252|PAPS1	Bifunctional 3-phosphoadenosine 5-phosphosulfate synthase 1	1,97	0,002
P40121-2|CAPG	Macrophage-capping protein	1,95	0,003
Q92599-3|SEPT8	Septin-8	1,91	0,003
Q9UJW0-2|DCTN4	Dynactin subunit 4	1,91	1E-04
O60547-2|GMDS	GDP-mannose 4,6 dehydratase	1,89	0,003
Q99729-3|ROAA	Heterogeneous nuclear ribonucleoprotein A/B	1,88	3E-07
Q8WUM4|PDC6I	Programmed cell death 6-interacting protein	1,86	7E-07
P06493|CDK1	Cyclin-dependent kinase 1	1,83	1E-04
P35244|RFA3	Replication protein A	1,72	1E-04
Q9GZZ1|NAA50	N-alpha-acetyltransferase 50	1,66	1E-04
Q9UBT2-2|SAE2	Isoform 2 of SUMO-activating enzyme subunit 2	1,64	3E-04
Q9NUU7|DD19A	ATP-dependent RNA helicase	1,62	2E-05
P43243|MATR3	Matrin-3	1,58	1E-04
P46926|GNPI1	Glucosamine-6-phosphate isomerase	1,53	5E-04
P30447|1A23	HLA class I histocompatibility antigen	1,29	9E-08

Supplementary Methods

Crosslinking of the tRNA^{Pro} half to crude HeLa ribosomes

To identify the ribosomal binding site and potential protein interactors of the tRNA^{Pro} 5' half *via* specific crosslinking, a 3'biotinylated tRNA^{Pro} half carrying three photo-reactive 4-thiouridine residues at positions 4, 13 and 27 was used (*Microsynth*). A 3' biotinylated tRNA^{Pro} half without 4-thio-U modifications was used as a negative control. Each crosslink reaction contained 1 µl of 100 µM tRNA^{Pro} half, 14 µl HeLa cell lysate and 4 µl water and was incubated for 30 min at 35°C. Subsequently, UV crosslink was performed for 10 min at 366 nm on ice. To enrich for ribosomes samples were subjected to ultracentrifugation using an AT140 rotor for 1.5 h at 200,000 xg. The pellet was resuspended in 50 µl denaturation buffer (10 mM Tris, pH 8; 0.1% SDS; 10 mM EDTA) and denaturation was performed for 10 min at 60°C. To purify proteins crosslinked to the biotinylated 4-thio-U-tRNA^{Pro} half, crosslinked samples were subjected to pull-down with Magnetic streptavidin beads (*Roche*). 50 µl of beads suspension were used for one sample containing around 300 pmol of biotinylated tRNA halves. Beads were washed tree times with 1 x BW buffer (10 mM Tris, pH 7; 1 mM EDTA; 2 M NaCl) and one time with 5 x SSC. In each washing step the supernatant was removed using a magnetic rack. In the final washing step, the suspension was aliquoted to the required number of tubes for following binding reaction, and the supernatant was removed the same way as before. Each binding reaction in a total volume of 200 µl contained 50 µl 20 x SSC + 0.4% Triton (*Sigma Aldrich*), crosslinked sample, 1 µl of RNase inhibitor RNasine (*Promega*). Binding was performed for 30 min at room temperature on a tube roller mixer. Unbound components were removed during multiple washing steps: (I) beads were washed for 10 min with 1 ml of washing buffer I (75 mM Hepes, pH 7.3, 150 mM NaCl, 0.1% Triton); (II) 5 min with 0.5 ml washing

buffer I, (III) 10 min with 0.5 ml washing buffer II (75 mM Hepes, pH 7.3, 500 mM NaCl, 0.1% Triton), (IV) 5 min with 0.5 ml washing buffer II, (V-VII) 10 min with 1 ml, 0.5 ml, and then 0.25 ml washing buffer III (75 mM Hepes, pH 7.5, 150 mM NaCl). All washing steps were performed at room temperature on a tube roller mixer. LS-MS analysis was performed on the purified beads.

RNase digestion of ProTiP

RNase digestions were performed on RNA isolated after *in vitro* translation with CHO cell lysate in the presence of [^{35}S]Met and tRNA^{Pro} half. To deacylate charged [^{35}S]Met-tRNA^{Met}, *in vitro* translation reactions were boiled in Laemmli buffer at 95 ºC for 5 min and then subjected to phenol-chloroform RNA extraction. Under these conditions the ester bond of [^{35}S]Met-tRNA^{Met} is hydrolyzed while the [^{35}S]Met-labeled ProTiP remains intact. RNA isolated from one *in vitro* translation reaction was resupended in 2.6 µl of water. For RNase A digestion, 1 µl of RNA was incubated with 2 µl AP buffer (7 M urea in 1x TBE) and 1 µl of 1-10 ng/µl RNaseA (*Thermo Scientific*) at 50 ºC for 10 min. For RNase T1 digestion, 1 µl of RNA was incubated first with 2 µl AP buffer at 95ºC for 2 min. Then 1 µl of 20-40 U/µl RNaseT1 (*Thermo Scientific*) was added and the reaction was incubated for 15 min on ice. After digestion, reactions were boiled for 2 min at 95 ºC in RNA loading dye and separated on a 12 % polyacrylamide sequencing gel. After electrophoresis the gel was vacuum-dried at 70°C for 2 h and exposed to a phosphorimager screen.

Supplementary References

[1] Hackl M, Jakobi T, Blom J, et al. Next-generation sequencing of the Chinese hamster ovary microRNA transcriptome: Identification, annotation and profiling of microRNAs as targets for cellular engineering. J. Biotechn. 2011; 153:62-75.

[2] Gerstl MP, Hackl M, Graf AB, et al. Prediction of transcribed PIWI-interacting RNAs from CHO RNAseq data. J. Biotechn. 2013; 166:51-57.

[3] Klinge S, Voigts-Hoffmann F, Leibundgut M, et al.Crystal structure of the eukaryotic 60S ribosomal subunit in complex with initiation factor 6. Science 2011; **334**:941-948.
